# Bioinspired Macrophage‐Camouflaged Oxygen‐Self‐Supplying Nanoplatform for Precision Synergistic Ablation of Breast Cancer

**DOI:** 10.1049/nbt2/4933996

**Published:** 2026-07-29

**Authors:** Yunwen Sun, Xun Gao, Juan Gao, Jiajia Chang, Xiaoqi Tan, Yue Yu, Yongping Lu, Da Huo

**Affiliations:** ^1^ The First Clinical Medical College, Nanjing Medical University, Nanjing 211169, China, njmu.edu.cn; ^2^ Department of Pharmaceutics, Nanjing Medical University, Nanjing 211169, China, njmu.edu.cn; ^3^ Guangyuan Central Hospital, Guangyuan 628000, China

**Keywords:** breast cancer, IrO_2_, M1 macrophage membrane, photodynamic therapy, photothermal therapy

## Abstract

Breast cancer remains one of the most prevalent and life‐threatening malignancies affecting women globally, and the development of precision therapeutic platforms capable of overcoming the inherent limitations of conventional monotherapies is a pressing clinical imperative. Here, we report the design, synthesis, and evaluation of a macrophage membrane‐camouflaged IrO_2_‐IR808 nanoplatform (IrO_2_‐IR808@M1) that achieves precise and synergistic tumor ablation through the cooperative action of photothermal/photodynamic therapy (PDT). The IrO_2_ nanocore provides efficient light‐to‐heat conversion and catalase‐like activity that locally generates oxygen to sustain PDT efficacy in the hypoxic tumor microenvironment, while the covalently conjugated near‐infrared (NIR) photosensitizer IR808 provides complementary NIR absorption and reactive oxygen species (ROS) generation. Under 808 nm NIR irradiation, IrO_2_‐IR808@M1 achieves rapid and reproducible temperature elevation with a high photothermal conversion efficiency (PCE) of 55.22%, translating into potent concentration‐dependent cytotoxicity in MCF‐7 breast cancer cells and pronounced tumor growth suppression in syngeneic 4T1 murine models without discernible systemic toxicity. The M1 macrophage membrane camouflage confers extended systemic circulation, enhanced colloidal stability, and strengthened tumor selectivity through immune‐mimetic surface interactions. This bioinspired, self‐oxygen‐supplying nanoplatform establishes a concise and translatable blueprint for precision phototherapy in breast cancer, with implications for broader application across solid tumor indications.

## 1. Introduction

Breast cancer is the most frequently diagnosed malignancy and the leading cause of cancer‐related mortality in women worldwide, accounting for over 2.3 million new cases and approximately 685,000 deaths annually, according to GLOBOCAN 2020 estimates [1–3]. Despite substantial advances in surgery, chemotherapy, hormone therapy, and targeted biologics, the clinical management of breast cancer, particularly triple‐negative and advanced‐stage subtypes, remains profoundly challenging. Conventional therapeutic modalities are frequently undermined by insufficient tumor selectivity, dose‐limiting systemic toxicity, and the emergence of multidrug resistance, collectively constraining achievable therapeutic windows and long‐term outcomes [4, 5]. In this context, light‐activated therapies have attracted considerable attention as spatiotemporally controllable alternatives. Photothermal therapy (PTT), driven by near‐infrared (NIR) light‐absorbing agents, enables highly localized cytolysis through the conversion of photonic energy into cytotoxic heat; however, PTT alone may yield incomplete tumor ablation, particularly at the tumor periphery, and its durability is inherently limited by thermal tolerance and heat dissipation [6–8]. Beyond direct cytolysis, the photothermal effect has been shown to enable controlled drug release from the carrier and to synergize with immune checkpoint regulation by augmenting T‐cell activation and antitumor immune responses, providing additional therapeutic dimensions when integrated with rationally designed nanoplatforms [6–9]. Photodynamic therapy (PDT) complements PTT by generating reactive oxygen species (ROS) that induce oxidative damage across a broader cellular landscape, yet PDT efficacy is critically constrained by the availability of molecular oxygen, rendering it particularly vulnerable in the characteristically hypoxic tumor microenvironment [10–13]. Integrating PTT and PDT within a single, rationally designed nanoplatform, therefore, offers a compelling strategy to overcome these individual limitations by synchronizing thermal and oxidative cytotoxic mechanisms, achieving additive or synergistic tumor ablation that neither modality can accomplish independently [14–16]. Furthermore, the photothermal effect enables controlled drug release from the carrier, and it synergizes with immune checkpoint therapy to enhance the T‐cell‐mediated antitumor immune response [17].

**Scheme 1:**
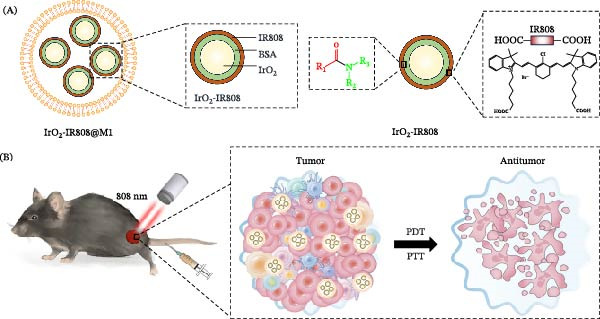
(A) Synthesis of IrO_2_‐IR808@M1 and (B) the mechanism of photothermal and photodynamic therapy to enhance the efficacy of cancer eradication.

Here, we report the rational design and systematic evaluation of an IrO_2_‐IR808 hybrid nanoplatform camouflaged with an M1‐polarized macrophage membrane (IrO_2_‐IR808@M1) as a bioinspired, oxygen‐self‐supplying phototherapeutic agent for precision breast cancer treatment. IrO_2_ was selected as the nanocore material on account of its well‐documented high photothermal conversion efficiency (PCE), excellent photostability under prolonged NIR exposure, and intrinsic catalase‐like enzymatic activity, wherein the latter enables the catalytic decomposition of endogenous hydrogen peroxide (H_2_O_2_) within the tumor microenvironment to locally generate molecular oxygen, thereby continuously replenishing the oxygen supply required for sustained PDT efficacy [12, 18, 19]. The NIR‐active photosensitizer IR808 was covalently conjugated to the BSA‐functionalized IrO_2_ surface via amide bond formation, providing complementary NIR absorption and ROS‐generating capacity for synergistic PTT‐PDT action [20]. To further advance the value of the nanoplatform, the IrO_2_‐IR808 hybrid nanoparticles were camouflaged with M1‐polarized macrophage membranes harvested from LPS/IFN‐γ‐activated RAW264.7 macrophages. Critically, the selection of an M1‐polarized membrane, rather than the M0 (naïve) or M2 (alternatively activated, protumorigenic) phenotype, is dictated by the distinct surface proteome of pro‐inflammatory macrophages. Specifically, M1 membranes display abundant activation‐dependent receptors that mediate inflammatory tropism toward tumor lesions and homotypic engagement with tumor cells, whereas M0 membranes lack such activation‐induced tumor‐tropic ligands, and M2 membranes bear immunosuppressive and protumorigenic cues (e.g., CD206‐mediated tumor‐supportive signaling) that are counterproductive for antitumor delivery. M1 membrane coating, therefore, maximizes tumor‐targeting capacity while avoiding the inadvertent delivery of protumorigenic biological signals [21, 22]. This tripartite architecture integrates efficient NIR‐driven heating, sustained oxygen‐fueled ROS generation, and biomimetic tumor targeting into a unified nanoplatform. The synthesis, physicochemical characterization, and therapeutic evaluation of IrO_2_‐IR808@M1 are detailed herein, with a comprehensive demonstration of its potent synergistic PTT‐PDT effects both in vitro and in vivo (Scheme 1).

## 2. Results and Discussion

**Figure 1:**
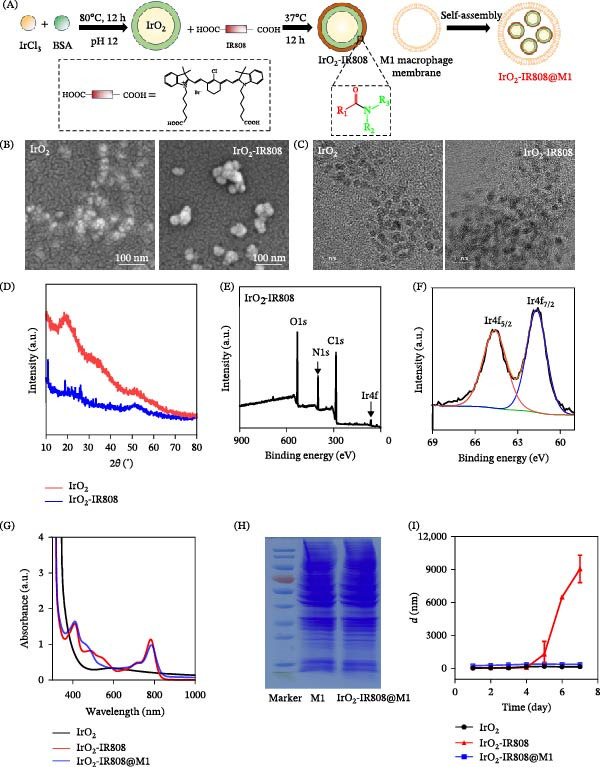
Synthesis and characterization of IrO_2_–IR808@M1. (A) Schematic of the preparation process. (B) SEM and (C) TEM images show spherical morphology and nanoclustered structure. (D) XRD with reflections indexed to the (101) and (211) planes of rutile‐phase IrO_2_ (JCPDS No. 43‐1019). (E, F) XPS and Ir4f spectra indicate surface modification. (G) UV–Vis spectra demonstrate enhanced NIR absorption. (H) SDS‐PAGE verifies membrane protein incorporation. (I) Hydrodynamic diameter measurements show improved stability after membrane cloaking.

IrO_2_ nanocrystals were synthesized via a BSA‐mediated biomineralization strategy under strongly alkaline conditions (pH~12, 80°C, 12 h), in which BSA served simultaneously as a reducing and capping agent, yielding a protein‐functionalized IrO_2_ nanocore with inherent colloidal stability and biocompatibility (Figure 1A). The photosensitizer IR808, which bears terminal carboxylic acid groups, was subsequently covalently conjugated to the surface‐exposed amine residues of BSA through carbodiimide‐mediated amide bond formation in the presence of DMAP, producing the IrO_2_‐IR808 hybrid with stable dye loading confirmed by UV–Vis spectroscopy. The final nanoplatform was assembled by membrane fusion: M1‐polarized macrophage membranes, harvested from RAW264.7 cells activated by co‐stimulation with LPS (100 ng/mL) and IFN‐γ (20 ng/mL) for 24 h, were isolated by hypotonic lysis and differential centrifugation and then coextruded with IrO_2_‐IR808 through a 200 nm polycarbonate membrane to yield uniformly sized, membrane‐cloaked IrO_2_‐IR808@M1 nanoparticles [23]. This stepwise modular assembly strategy affords precise compositional control at each stage while preserving the structural integrity of the biological membrane and the photophysical properties of both the IrO_2_ core and the IR808 photosensitizer.

The morphological and structural characteristics of the nanoplatform were systematically interrogated by complementary physicochemical techniques. Scanning electron microscopy (SEM, Figure 1B) and transmission electron microscopy (TEM, Figure 1C) revealed that both IrO_2_ and IrO_2_‐IR808 adopted a well‐defined spherical architecture with a narrow size dispersity. Notably, TEM imaging at higher magnification resolved the presence of ultrafine nanoclusters of ~2 nm within the IrO_2_‐IR808 composite, indicative of a hierarchical nanoclustered substructure that maximizes the accessible surface area and promotes intimate interfacial contact between the IrO_2_ core and the conjugated IR808 molecules, which collectively are anticipated to enhance the optical absorption cross‐section and photon‐to‐heat transduction efficiency. X‐ray diffraction (XRD) analysis confirmed the crystalline nature of the IrO_2_ phase, with diffraction reflections at ~34° and 53° corresponding to the (101) and (211) crystal planes of rutile‐phase IrO_2_, respectively (Figure 1D and Figure S1A). The X‐ray photoelectron spectroscopy (XPS) of IrO_2_ shows peaks of O1s, N1s, C1s, and Ir4f (Figure S1B). The characteristic peaks at 61.7 and 64.7 eV correspond to 4 f 7/2 and 4 f 5/2 of Ir^4+^, respectively (Figure S1C). Combined with the XRD results, these XPS data collectively verified the successful fabrication of IrO_2_. XPS survey scans of IrO_2_–IR808 identified characteristic photoelectron signals corresponding to O1s, N1s, C1s, and Ir4f core levels (Figure 1E), confirming successful BSA functionalization and IR808 conjugation. High‐resolution XPS analysis of the Ir4f region revealed a discernible positive binding energy shift in both the Ir4f_5_/_2_ and Ir4f_7_/_2_ peaks upon BSA/IR808 functionalization relative to pristine IrO_2_ (Figure 1F), reflecting electron density redistribution at the iridium surface and increased electrophilic character, consistent with the formation of Ir–N coordination bonds between the IrO_2_ surface and the amine residues of BSA and suggestive of an intimate electronic coupling between the organic photosensitizer layer and the inorganic nanocore.

Dynamic light scattering (DLS) measurements quantified the hydrodynamic size evolution across the three fabrication stages: IrO_2_‐IR808 exhibited a hydrodynamic diameter of 49.47 nm, which increased substantially to 242.35 nm following M1 macrophage membrane encapsulation, consistent with the addition of a continuous lipid bilayer and associated membrane‐anchored protein components around the nanocore (Figure S2). The surface zeta potential remained negative throughout, ranging from −20.55 mV for IrO_2_‐IR808 to −10.32 mV for IrO_2_‐IR808@M1, indicating preserved electrostatic colloidal stability despite the incorporation of the biomimetic membrane shell. Successful transfer and retention of M1 macrophage membrane proteins onto the nanoplatform surface were confirmed by SDS‐PAGE, which revealed a protein banding pattern in IrO_2_‐IR808@M1 closely recapitulating that of the parental M1 membrane fraction and by the detection of the M1‐specific surface marker CD86 (Figure 1H and Figure S3), validating the biological fidelity of the membrane cloaking process. Concurrent monitoring of hydrodynamic diameter, polydispersity index (PDI = 0.1573), and ζ‐potential of IrO_2_‐IR808@M1 over the same 7‐day window revealed essentially no fluctuation in any of these three parameters, collectively confirming that the M1 membrane shell affords colloidal stabilization rather than acting as an aggregation‐inducing layer (Figure S4). UV–Vis absorption spectroscopy demonstrated that IrO_2_‐IR808 exhibited a pronounced and red‐shifted NIR absorption peak at 781 nm compared to the 588 nm peak of bare IrO_2_ (Figure 1G), confirming successful IR808 incorporation and the anticipated enhancement of NIR light harvesting capacity. Longitudinal DLS measurements over 7 days confirmed that IrO_2_‐IR808@M1 maintained stable hydrodynamic dimensions with minimal aggregation, in marked contrast to IrO_2_‐IR808 alone, which showed progressive size increases indicative of aggregation (Figure 1I), demonstrating that membrane cloaking substantially improves colloidal stability under physiologically relevant conditions.

TMB colorimetric assays were performed to verify the catalase‐like activity of the nanomaterials. Both IrO_2_ and IrO_2_‐IR808@M1 catalyzed H_2_O_2_ decomposition into H_2_O and O_2_, accompanied by TMB oxidation to blue oxTMB. Quantitative absorbance readings at 652 nm confirmed strong catalase‐mimicking activity for both samples (Figure S5A). Visible bubbles also appeared after incubating IrO_2_ or IrO_2_‐IR808@M1 with H_2_O_2_, directly demonstrating the H_2_O_2_ breakdown (Figure S5B). A [Ru(dpp)_3_]Cl_2_ fluorescent probe was further used to detect O_2_ production from H_2_O_2_ degradation. The control group exhibited intense red fluorescence, whereas the IrO_2_ and IrO_2_‐IR808@M1 groups showed drastically weakened fluorescent signals (Figure S4C). Collectively, these results verify that the self‐oxygen‐supplying nanoplatform possesses catalase‐mimicking activity and efficiently decomposes H_2_O_2_ to generate O_2_.

**Figure 2:**
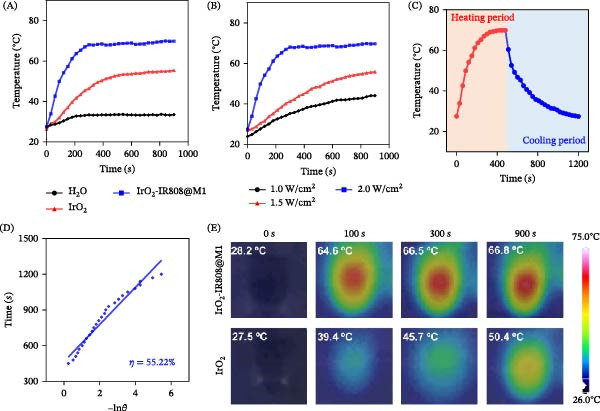
Photothermal properties of IrO_2_‐IR808@M1 under NIR irradiation. (A) Temperature profiles for H_2_O, IrO_2_, and IrO_2_‐IR808@M1. (B) Power‐dependent heating curves. (C) Heating/cooling cycle demonstrates thermal stability. (D) Cooling kinetics show linear heat dissipation. (E) Thermal images visualize efficient photothermal conversion.

The photothermal performance of IrO_2_‐IR808@M1 was comprehensively evaluated under 808 nm NIR irradiation at a power density of 2.0 W/cm^2^. Upon continuous irradiation for 15 min, IrO_2_‐IR808@M1 exhibited a rapid and pronounced temperature elevation, reaching a steady‐state temperature of ~69.8°C, a value substantially exceeding that achieved by IrO_2_ alone (~55.4°C) under identical conditions and representing a temperature increase of more than 40°C above ambient conditions (Figure 2A). This superior thermal response reflects the synergistic contribution of IR808’s strong NIR absorption to the intrinsic photothermal capacity of the IrO_2_ nanocore, as well as the improved dispersion stability conferred by the macrophage membrane coating, which prevents nanoparticle aggregation and maintains optimal optical cross‐section. Power‐dependent heating experiments demonstrated that the temperature elevation scaled predictably with irradiation power density across the range of 1.0–2.0 W/cm^2^ (Figure 2B and Figure S6), confirming that the thermal output of IrO_2_‐IR808@M1 can be rationally modulated by adjusting irradiation parameters to achieve target therapeutic temperatures while minimizing off‐target thermal damage to the surrounding normal tissue. Heating–cooling cycle measurements demonstrated excellent thermal reproducibility and structural stability (Figure 2C), with the nanoplatform retaining its photothermal performance across repeated irradiation cycles, a critical prerequisite for multisession phototherapy regimens.

The PCE of IrO_2_‐IR808@M1 was quantitatively determined from the cooling kinetics following cessation of NIR irradiation. Analysis of the linear relationship between cooling time and the negative natural logarithm of the dimensionless temperature driving force (−ln *θ*) yielded a characteristic thermal time constant (τs), from which a PCE of 55.22% was calculated (Figure 2D), a value that compares favorably with state‐of‐the‐art photothermal agents reported in the literature and reflects highly efficient photon‐to‐heat energy transduction within the nanoplatform. Infrared thermal imaging further corroborated these findings, visually capturing the rapid and spatially homogeneous temperature rise across the IrO_2_‐IR808@M1 solution relative to IrO_2_ alone at matched time points (Figure 2E). The superior PCE of IrO_2_‐IR808@M1 relative to IrO_2_ alone is mechanistically attributed to the synergistic optical absorption contributions of the IrO_2_ nanocore and IR808, whose absorption bands overlap constructively in the NIR window, as well as to the enhanced dispersibility afforded by the macrophage membrane, which maximizes the effective optical path length and minimizes scattering losses. The linearity and predictability of the cooling curve further indicate that heat dissipation in IrO_2_‐IR808@M1 follows first‐order kinetics, facilitating the precise pharmacological modeling of therapeutic temperature windows and enabling rational scheduling of combined PTT‐PDT treatment protocols.

**Figure 3:**
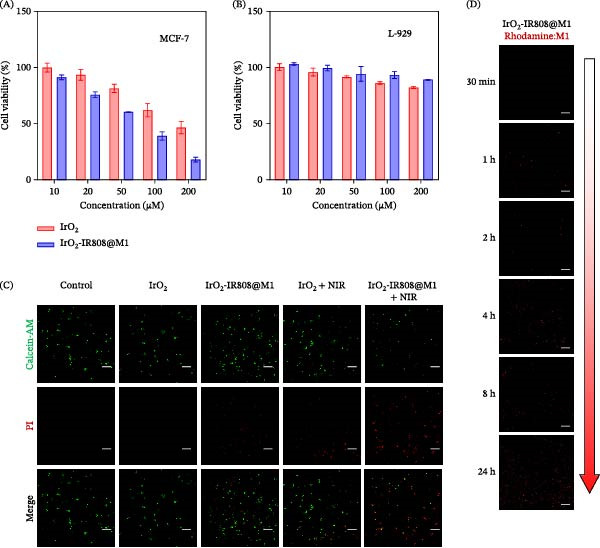
In vitro cytotoxicity and cellular uptake of IrO_2_–IR808@M1. (A) MCF‐7 cell viability after treatment and NIR irradiation. (B) L929 fibroblast viability confirms selectivity. (C) Fluorescence microscopy shows maximal cell death with combined treatment. (D) Time‐dependent uptake of rhodamine B‐labeled IrO_2_‐IR808@M1 in MCF‐7 cells. Scale bar: 200 μm.

The cytotoxic efficacy and tumor selectivity of IrO_2_‐IR808@M1 were systematically assessed in MCF‐7 human breast cancer cells and L929 murine fibroblasts as a normal cell reference. In MCF‐7 cells, both IrO_2_ and IrO_2_‐IR808@M1 induced concentration‐dependent reductions in cell viability under 808 nm NIR irradiation (2.0 W/cm^2^, 10 min), with IrO_2_‐IR808@M1 exhibiting markedly enhanced cytotoxicity relative to IrO_2_ alone across all tested concentrations (Figure 3A). At the highest tested concentration of 200 µM, IrO_2_‐IR808@M1 reduced MCF‐7 viability to ~18.1%, compared to 46.6% for IrO_2_ alone. A more than 2.5‐fold improvement in cancer cell killing efficacy was directly attributable to the additive contribution of IR808‐mediated PDT‐driven ROS generation to the IrO_2_‐derived photothermal cytotoxicity, confirming the anticipated synergistic interaction between the two therapeutic modalities. In marked contrast, L929 fibroblasts retained ≥83%–90% viability across the entire concentration range tested (10–200 µM) under both IrO_2_ and IrO_2_‐IR808@M1 treatments (Figure 3B), demonstrating a highly favorable therapeutic selectivity index and excellent biocompatibility toward nonmalignant cells. This differential cytotoxic response between cancer and normal cell lines is consistent with the elevated metabolic activity and altered redox homeostasis of malignant cells, rendering them selectively vulnerable to combined oxidative and thermal insults. Calcein‐AM/propidium iodide (PI) dual‐fluorescence live/dead staining provided orthogonal visual confirmation of these quantitative findings (Figure 3C): the IrO_2_‐IR808 + NIR treatment group exhibited the most pronounced and spatially uniform cell death, characterized by extensive PI‐positive (dead cell) fluorescence and near‐complete suppression of the Calcein‐AM (live cell) signal, whereas control, IrO_2_ alone, and IrO_2_‐IR808@M1 without NIR groups retained predominantly viable cell populations.

To directly evaluate the role of the M1 macrophage membrane in mediating cellular association and internalization, the membrane component of IrO_2_‐IR808@M1 was fluorescently labeled with rhodamine B, and time‐dependent intracellular accumulation was monitored in MCF‐7 cells by fluorescence microscopy over a period spanning from 30 min to 24 h (Figure 3D). Fluorescence signals within the MCF‐7 cell interior were detectable as early as 30 min postincubation and increased progressively with time, reaching robust intracellular accumulation by 24 h (Figure S7). This time‐dependent internalization profile confirms that the M1 macrophage membrane facilitates efficient and sustained cellular engagement, likely through a combination of membrane fusion, macropinocytosis, and receptor‐mediated endocytosis pathways enabled by the preserved surface proteins of the macrophage membrane, including adhesion molecules and pattern recognition receptors that promote interaction with tumor cells [22]. These results validate the functional contribution of the biomimetic membrane coating not only to colloidal stability but also to active cellular targeting, thereby ensuring sufficient intracellular drug accumulation to achieve therapeutically meaningful PTT‐PDT effects upon NIR activation.

**Figure 4:**
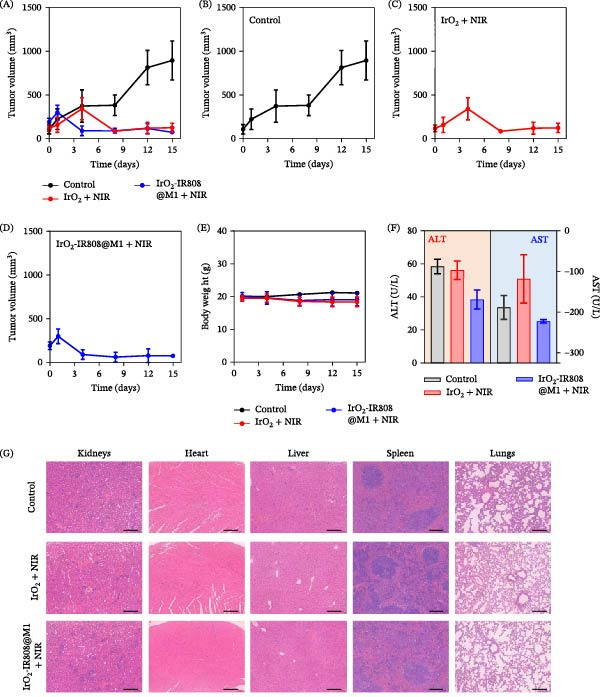
In vivo antitumor efficacy and biosafety of IrO_2_‐IR808@M1 in 4T1 tumor‐bearing mice. (A) Tumor growth curves for different treatments. (B–D) Representative tumor growth in each group. (E) Body weight monitoring. (F) Liver function tests posttreatment. (G) H&E staining of tumors and major organs. Scale bar: 200 μm.

The in vivo therapeutic efficacy of IrO_2_‐IR808@M1 was evaluated in female BALB/c mice bearing syngeneic 4T1 orthotopic breast tumors, a clinically relevant and immunocompetent model widely employed for the preclinical assessment of breast cancer nanomedicines [24]. When average tumor volumes reached ~100 mm^3^, mice were randomized into three groups (*n* = 3 per group) and administered 200 µL of PBS (control), IrO_2_ (100 µg/mL), or IrO_2_–IR808@M1 (100 µg/mL) via tail vein injection, followed by 808 nm laser irradiation (1.5 W/cm^2^, 10 min) at the tumor site 24 h postinjection; this treatment cycle was repeated every 3 days over a 15‐day observation period. IrO_2_‐IR808@M1 + NIR treatment produced the most pronounced and earliest onset of tumor suppression among all groups: significant tumor volume reduction was observable by day 1 posttreatment, and tumor growth remained durably suppressed throughout the 15‐day monitoring period (Figure 4A–D). By contrast, IrO_2_ + NIR induced a delayed and less complete response, with measurable tumor regression apparent only from day 4, while PBS‐control mice exhibited unimpeded tumor progression, collectively underscoring the therapeutic superiority conferred by the synergistic PTT‐PDT action and the tumor‐targeting enhancement provided by the macrophage membrane coating. Throughout the entire treatment period, the body weight remained stable across all groups with no significant intergroup differences (Figure 4E), indicating the absence of systemic toxicity. Comprehensive hematological profiling (Figure S8) and hepatic function assessment by serum alanine aminotransferase (ALT) and aspartate aminotransferase (AST) measurements (Figure 4F) confirmed that all key parameters remained within clinically normal reference ranges for IrO_2_‐IR808@M1‐treated animals. Histological examination of major organs including the kidney, heart, liver, spleen, and lung by hematoxylin and eosin (H&E) staining revealed no pathological abnormalities, inflammatory infiltrates, or tissue necrosis in any treatment group (Figure 4G), collectively establishing the excellent systemic biocompatibility and favorable safety profile of IrO_2_‐IR808@M1.

The robust and durable antitumor efficacy of IrO_2_‐IR808@M1 can be mechanistically attributed to three mutually reinforcing and temporally coordinated processes that collectively amplify therapeutic outcomes beyond what any single modality could achieve in isolation. First, high‐efficiency NIR‐driven photothermal heating by the IrO_2_ nanocore induces direct thermal cytolysis of tumor cells and creates a state of thermal vulnerability characterized by membrane disruption, mitochondrial dysfunction, and protein denaturation that sensitizes surviving cells to subsequent oxidative insults [25]. Second, IR808‐mediated Type II PDT generates cytotoxic singlet oxygen (^1^O_2_) and other ROS species that independently induce apoptotic and necrotic cell death through oxidative damage to lipids, proteins, and nucleic acids, extending cytotoxicity beyond the spatial and temporal boundaries of the thermal ablation zone [26]. Third, the catalase‐like enzymatic activity of IrO_2_ catalyzes the decomposition of endogenous tumor H_2_O_2_ into molecular oxygen, locally alleviating the hypoxic tumor microenvironment and continuously replenishing the oxygen substrate required for sustained IR808‐mediated PDT, a self‐supplying oxygen mechanism that circumvents the principal limitation of conventional PDT in solid tumors [27]. Furthermore, PTT‐induced elevation of tumor blood perfusion and vascular permeability may transiently augment oxygen delivery to the irradiated region, creating a positive feedback loop that further amplifies the PDT efficacy [28]. These findings suggest that deliberate temporal optimization of the PTT‐PDT activation sequence, for instance, preheating to enhance tumor oxygenation before maximal PDT activation, represents a rational strategy to further maximize therapeutic synergy while minimizing collateral photothermal damage to surrounding normal tissue.

In summary, we have successfully designed, synthesized, and evaluated IrO_2_‐IR808@M1, a bioinspired macrophage membrane‐camouflaged nanoplatform that integrates oxygen‐self‐supplying photocatalytic activity, high‐efficiency NIR photothermal conversion (PCE = 55.22%), and biomimetic tumor targeting into a unified therapeutic architecture for precision breast cancer ablation. The synergistic coupling of PTT and PDT within this platform achieves markedly superior tumor cell killing compared to either modality alone, as demonstrated by the reduction of MCF‐7 viability to 18.1% in vitro and by durable tumor growth suppression in syngeneic 4T1 murine models in vivo, all in the absence of discernible systemic toxicity, as confirmed by hematological, hepatic, and histological assessments. The M1 macrophage membrane camouflage is identified as a critical functional component, contributing to improved colloidal stability, prolonged systemic circulation, and enhanced tumor‐selective accumulation through immune‐mimetic surface interactions. These results establish IrO_2_‐IR808@M1 as a robust and translatable platform for precision phototherapy and motivate future investigations into the immunological consequences of M1 membrane‐mediated tumor engagement, the optimization of PTT‐PDT temporal sequencing, and the extension of this bioinspired design logic to additional solid tumor indications.

## Funding

This work was supported by the National Natural Science Foundation of China (Grants 12347102 and 32371447).

## Conflicts of Interest

The authors declare no conflicts of interest.

## Supporting Information

Additional supporting information can be found online in the Supporting Information section.

## Supporting information


**Supporting Information** The Supporting Information accompanying this article provides the detailed experimental protocols for the synthesis and characterization of IrO2, IrO2‐IR808, and IrO2‐IR808@M1, together with the methods used for M1 macrophage polarization, catalase‐mimicking activity validation, photothermal evaluation, in vitro cytotoxicity/uptake assays, and the in vivo therapeutic performance and hematological safety profiling in tumor‐bearing mice.

## Data Availability

The data supporting this article have been included as presented in the main text or Supporting Information.
