# Supplementary material for: Bioinspired Macrophage‐Camouflaged Oxygen‐Self‐Supplying Nanoplatform for Precision Synergistic Ablation of Breast Cancer
Source: IET Nanobiotechnol. 2026 Jul 29;2026:4933996. doi: 10.1049/nbt2/4933996 (PMC13420270; doi:10.1049/nbt2/4933996)
Supplement: Supplementary file 1 — Supporting Information The Supporting Information accompanying this article provides the detailed experimental protocols for the synthesis and characterization of IrO2, IrO2‐IR808, and IrO2‐IR808@M1, together with the methods used for M1 macrophage polarization, catalase‐mimicking activity validation, photothermal evaluation, in vitro cytotoxicity/uptake assays, and the in vivo therapeutic performance and hematological safety profiling in tumor‐bearing mice. [file NBT2-2026-4933996-s001.docx]

Supporting Information

**Bioinspired Macrophage-Camouflaged Oxygen-Self-Supplying Nanoplatform for Precision Synergistic Ablation of Breast Cancer**

*Yunwen Sun^+^, Xun Gao^+^, Juan Gao, Jiajia Chang, Xiaoqi Tan, Yue Yu, Yongping Lu, Da Huo**

Y. Sun

The First Clinical Medical College, Nanjing Medical University, Nanjing 211169, P. R. China.

X. Gao, J. Gao, J. Chang, X. Tan, D. Huo.

Department of Pharmaceutics, Nanjing Medical University, Nanjing 211169, P. R. China.

E-mail addresses: [huoda@njmu.edu.cn](mailto:huoda@njmu.edu.cn)

Y. Yu, Y. Lu

Guangyuan Central Hospital, Guangyuan 628000, P. R. China.

**Materials and Methods**

*Materials.*

Bovine serum albumin (BSA) and Tris-HCl were purchased from BioFroxx (Einhausen, Germany). Iridium trichloride (IrCl3), Sodium hydroxide (NaOH), sodium chloride (NaCl), Magnesium chloride(MgCl2), Potassium chloride (KCl), and 1- (5-Carboxypentyl) -2- (2- (3- (2- (1- (5-carboxypentyl) -3,3-dimethylindolin-2-ylidene) ethylidene)-2-chlorocyclohex-1-en-1-yl)vinyl)-3,3-dimethyl-3H-indol-1-ium bromide (IR808) were purchased from Macklin Biochemical Technology Co., Ltd (Shanghai, China). N, N-dimethyl 4-pyridinamine (DMAP) was purchased from Aladdin Biochemical Technology Co., Ltd (Shanghai, China). Lipopolysaccharide (LPS), Recombinant Human IFN-γ Protein (IFN-γ), InStabTM Protease Cocktail (EDTA-free), and Calcein-AM/PI Double Stain Kit Calcein-AM/PI were purchased from Shanghai Yuanye Biotechnology Co., Ltd (Shanghai, China). Phosphate Buffered Saline (PBS) was purchased from Thermo Fisher Scientific Co., Ltd (Shanghai, China). BCA protein assay kits, SDS-PAGE Gel Preparation Kit, Coomassie Blue Staining Kit, Prestained Color Protein Marker, SDS-PAGE Sample Loading Buffer, and Cell Counting Kit-8 were purchased from Beyotime Co., Ltd (Shanghai, China). PE-Cyanine7 Anti-Mouse CD206 Rabbit Recombinant Antibody, and APC Anti-Mouse CD86 were purchased from Proteintech Group Co., Ltd (Wuhan, China). Trypsin-EDTA was purchased from Gibco Co., Ltd (New York, USA)

*Synthesis of* *IrO_2_-IR808.*

**Synthesis of IrO_2:_** The IrO_2_ nanoparticles were obtained via a biomineralization process. 8 mL BSA (18.8 mg/mL) was incubated with 2.0 mL IrCl₃ (50 mM) for 30 min under vigorous stirring. The solution was adjusted to a pH 12 with 2.0 M NaOH. After reacting at 80 ℃ for 12 h, the obtained IrO_2_ nanoparticles were dialyzed for 24 h in deionized water to remove the excess precursors (MWCO=14,000 Da).

**Synthesis of IrO_2_-IR808:** IrO_2_-IR808 was achieved through an amideation reaction. 1 mL IR808 (1.0 mM) and 1 mL DMAP (0.1 M) were added to the 1 mL IrO_2_ (1.0 mM). The mixture was stirred at 37 ℃ for 12 h. The solution was centrifuged at 12,000 rpm for 20 min and resuspended in Tris-HCl buffer (10 mM Tris-HCl, 100 mM NaCl, 5 mM MgCl_2_, pH 7.4). The centrifugation was repeated three times to obtain purified IrO_2_-IR808.

*Synthesis of IrO_2_-IR808@M1.*

**M1 macrophages membranes:** In the presence of 100 ng/mL LPS and 20 ng/mL IFN-γ, Raw264.7 cells were induced to undergo polarization for 24 h to obtain M1 macrophages. After washing twice with cold PBS, M1 macrophages were collected using a cell scraper. Centrifuge at 800g for 5 min, resuspend the precipitate in hypotonic lysis buffer. Vortex the solution evenly and incubate at ice for 5 mi. The suspension was treated with an ultrasonic crusher (300W, 40kHz) for 5 min to ensure that the cells were fully disrupted. Centrifuge at 16900g for 30 min at 4℃ to obtain the M1 macrophage membranes. Finally, the cell membranes were stored at -80℃. Hypotonic lysis buffer: 2 mM MgCl_2_, 10 mM KCl, 20 mM Tris-HCl, 1 μL proteinase inhibitor without EDTA.

**Synthesis of IrO_2_-IR808@M1**: IrO_2_-IR808@M1 was obtained by the extrusion method. The M1 macrophage membrane was mixed uniformly with IrO_2_-IR808 at a ratio of 1:1 (w/w). After uniformly mixing the membrane with the nanoparticles by ultrasound, the solution was incubated at 4℃ overnight to form a stable M1 macrophage membrane encapsulating the IrO_2_-IR808 structure. The suspension was extruded through a lipid particle separator with a polycarbonate membrane pore size of 200 nm to obtain uniformly sized IrO_2_-IR808@M1, which was then stored at 4 ℃.

*Characterization.*

Scanning electron microscopy (SEM, Gemini SEM 300, Zeiss, Germany) and transmission electron microscope (TEM, JEM-F200, JEOL, Japan) were used to examine the surface morphology of IrO_2_ and IrO_2_-IR808. X-ray diffraction (XRD, D8 Advance, Bruker, Germany) was performed to test the phase identification of IrO_2_ and IrO_2_-IR808. X-ray Photoelectron Spectroscopy (XPS, K-Alpha, Thermo Scientific, USA) was performed to analyze the elemental composition, chemical valence and electronic state of the surfaces of IrO_2_ and IrO_2_-IR808. Dynamic light scattering (DLS) was performed to measure the hydrodynamic diameter of IrO_2,_ IrO₂-IR808 and IrO₂-IR808@M1. Zeta-potential measurements were performed using the same instrument.UV-visible spectrophotometer (P4, MAPADA, China) was performed to conduct a qualitative analysis of IrO_2_ and IrO_2_-IR808.

The BCA protein assay kits was used to assess the concentration of membrane proteins in M1 macrophages. The concentrations of the BCA protein standard solutions are 0, 0.025, 0.05, 0.1, 0.2, 0.3, 0.4, and 0.5 mg/ml. 2 μL M1, IrO_2_-IR808@M1 are added to 18 μL standard dilution solution. After adding 200 μL BCA working solution to each group, incubate at 37℃ for 30 min. The absorbance of the solution at 562nm was measured by microplate reader (ReadMax 1200, Shanpu, China). A standard curve was plotted based on the absorbance values of standard samples, and the protein concentration in M1, IrO_2_-IR808@M1 was calculated. 40 μg protein sample, 6 μL 5×loading buffer, add buffer to 30 μL for form the sample solution, and mix evenly. The sample solution was incubated at 100 ℃ for 5 min to protein denaturation. 10% SDS-polyacrylamide gel electrophoresis gradient gel for 90 min at 120 V was used to separate the proteins of M1 and IrO_2_-IR808@M1 After staining the gel with the coomassie blue staining at room temperature for 2 h, the gel was de-colored at room temperature for 12 h. The protein bands were evaluated using Gel imaging system (GenoSens 2000, Qinxiang Science Instrument, China).

*M1 polarization of RAW264.7 macrophages and verification by flow cytometry*

RAW264.7 murine macrophages were maintained in DMEM supplemented with 10 % fetal bovine serum (FBS) and 1 % penicillin–streptomycin in a humidified incubator (37 °C, 5 % CO₂). For M1 polarization, cells were seeded at 1 × 10⁶ cells per well in 6-well plates and co-stimulated with lipopolysaccharide (LPS, 100 ng mL⁻¹) and recombinant murine interferon-γ (IFN-γ, 20 ng mL⁻¹) for 24 h. Unstimulated cells served as the M0 baseline control. After stimulation, cells were harvested by gentle scraping, washed twice with ice-cold PBS containing 2 % FBS, and incubated with Fc-block (anti-CD16/32) for 10 min on ice to suppress non-specific binding. Cells were then surface-stained with PE-conjugated anti-mouse CD86 antibody and APC-conjugated anti-mouse CD206 antibody for 30 min on ice in the dark (concentrations as recommended by the manufacturer; please insert clone numbers and vendor information). After two washes with FACS buffer, cells were resuspended in PBS containing 1 % paraformaldehyde and analyzed on a BD FACS Calibur flow cytometer; ≥ 1 × 10⁴ events were acquired per sample. Compensation was performed using single-stain controls, and gating strategy followed forward/side-scatter (FSC/SSC) singlet selection. Data analysis was performed in FlowJo v10. Three independent biological replicates were performed.

*Photothermal Properties of IrO_2_-IR808@M1.*

The photothermal performance of IrO₂-IR808@M1 was characterized using a near-infrared thermal imaging system (Ti401 PRO, Fluke, USA). 500 μL H₂O, IrO₂ (200 μM), and IrO₂-IR808@M1 (200 μM) were irradiated with an 808 nm laser at a power density of 2.0 W/cm² for 15 min, and the temperature of the solutions was recorded every 30 s. Furthermore, the temperature variations of the system were measured under laser power densities of 1.0, 1.5, and 2.0 W/cm². The photothermal conversion efficiency of the IrO₂-IR808@M1 was also investigated. The IrO₂-IR808@M1 photothermal conversion efficiency was calculated using the following formula.

$$\eta=\frac{\mathrm{hs}\left( T_{\max}-T_{\mathrm{surr}} \right)-Q_{0}}{I(1-{10}^{-A808})}$$

$$Q_{0}=hS\left( T_{max,water}-T_{\mathrm{surr}} \right)$$

$$\tau_{s}=\frac{m_{d}C_{d}}{\mathrm{hS}}$$

$$t=-\tau_{s}ln\theta$$

$$\theta=\frac{T-T_{\mathrm{surr}}}{T_{\max}-T_{\mathrm{surr}}}$$

$\tau_{s}$: Characteristic thermal time constant. $T_{\max}$: Steady-state temperature. $T_{\mathrm{surr}}$: Ambient temperature. $T$: Real-time temperature. $m_{d}$: Mass of the solvent (H_2_O, 1 g). $C_{d}$: Heat capacity of the solvent (H_2_O, 4.2 J/g). $I$: Laser power (W). $A808$: Absorbance of IrO₂-IR808@M1 at 808 nm.

*Catalase-mimicking activity validation*

**TMB chromogenic assay**: IrO₂ or IrO₂-IR808@M1 (final nanoparticle concentration 50 µg mL⁻¹) was incubated with H₂O₂ (final concentration 1 mM) and TMB (final concentration 0.4 mM) in acetate buffer (pH 4.5) at 37 °C for 30 min. The reaction was terminated by addition of 2 M H₂SO₄, and absorbance at 652 nm was recorded on a microplate reader. Three independent measurements (n = 3) per group were performed; PBS only served as the control.

**Macroscopic gas-bubble evolution**: IrO₂ or IrO₂-IR808@M1 (200 µg mL⁻¹, 1 mL) was added to a 10 mM H₂O₂ aqueous solution in a transparent glass vial, and photographs were taken at fixed time points using a digital camera against a uniform white background; PBS + H₂O₂ served as the bubble-free control.

**[Ru(dpp)₃]Cl₂ phosphorescence quenching**: [Ru(dpp)₃]Cl₂ (1 µM) was co-incubated with IrO₂, IrO₂-IR808@M1, or PBS (control) in the presence of H₂O₂ (1 mM) for 30 min at 37 °C. Fluorescence images were acquired on a confocal laser scanning microscope (λ_ex = 455 nm; λ_em = 613 nm) with identical laser power, gain and acquisition parameters across groups. Decreased red phosphorescence intensity in the IrO₂ and IrO₂-IR808@M1 groups indicated local O₂ generation.

*Cell Culture.*

L929, MCF-7, 4T1 cells was obtained from the American Type Culture Collection (ATCC, MD, USA). The cells were cultured in DMEM/F12 medium, supplemented with 10% FBS (FBS, Gibco, USA) and 1% penicillin/streptomycin (P/S, Gibco, USA).

*Cell Counting Kit-8.*

CCK-8 assay quantitatively measured material cytotoxicity (cell viability) and verified its specific killing efficacy under near-infrared (NIR) light irradiation. L929 and MCF-7 cells were seeded in 96-well plates with 2×10^3^ cells per well and incubated for 24 h. Then L929 cells were incubated with IrO₂ or IrO₂-IR808 for 24 h at different concentrations. Notably, MCF-7 cells were initially incubated with IrO₂ or IrO₂-IR808@M1 for 8 h, and then irradiated with an 808 nm laser (2.0 W/cm^2^, 10 min). This experiment group continued to incubate for another 16 h. After incubation, all groups were washed with PBS three times, and 100 μL fresh medium was added. After that, 10 µL CCK-8 was added to each well and further incubated for another 1 h at 37 ℃. The absorbance at 450 nm was observed under a microplate reader. The cell viability of the experimental group was calculated based on the absorbance values of the control group.

*Live/Dead Cell Staining Assay.*

Calcein-AM/PI staining and fluorescence microscopy provided direct visualization of live and dead cells. The MCF-7 cells were seeded in 24-well plates with a density of 1×10^5^ cells each well and incubated for 24 h. Then, the cells were incubated with 200 μM IrO₂, IrO₂-IR808 for 24 h. Specifically, the NIR irradiation group was exposed to a 2.0 W/cm^2^ laser for 10 min at the 8 h. MCF-7 Cells were digested using 0.25% trypsin-EDTA and subsequently collected by centrifugation at 1200 rpm for 3 min. The cells were washed three times with 1×Assay Buffer to thoroughly remove residual esterase activity. The cells were resuspended in 1×Assay Buffer to a density of 1×10⁵ to 1×10⁶ cells/mL. 100 µL staining working solution, containing 2 µM Calcein-AM and 4.5 µM propidium iodide (PI), was added to 200 µL cell suspension, followed by incubation at 37 °C for 15 min. The cells were observed under a fluorescence microscope (DM IL LED, Leica, Germany).

*Internalization and IrO_2_-IR808 in Cancer Cells.*

Rhodamine staining combined with fluorescence microscopy was used to confirm the internalization of nanoparticles into the target cancer cells. The MCF-7 cells were seeded in 24-well plates with a density of 1×10^5^ cells each well and incubated for 24 h. Then, the cells were incubated with 200 μM IrO_2_-IR808@Rhodamine-M1 at 30 min, 1 h, 2 h, 4 h, 8 h, 12 h, and 24 h. The cells were washed three times with PBS, and observed under a fluorescence microscope (DM IL LED, Leica, Germany).

*Animal Model.*

Female BALB/c mice (5–6 weeks old, 20–25 g) were obtained from Chengdu Senwei Laboratory Animal Co., Ltd. The 4T1 tumor model was established by subcutaneous injection of 1 × 10⁶ 4T1 cells into the flank region of the outer thigh. All the animal operations conformed to the guidelines of the Animal Care Ethics Commission of Guangyuan Central Hospital (Animal Ethics No.: GYZXLLKD2026001).

*In Vivo Antitumor Efficacy Studies.*

When the average tumor volume reached 100 mm³, the mice were randomly divided into three groups (n=3) and administered via tail vein injection with 200 µL PBS, IrO₂ (100 µg/mL), or IrO₂-IR808@M1 (100 µg/mL). At 24 h post-injection, the tumor regions were irradiated with an 808 nm laser at a power density of 1.5 W/cm² for 10 min. The same laser treatment was repeated every three days. Throughout the 15-day treatment period, body weight was measured and tumor size was monitored every three days using a caliper. Tumor volume was calculated according to the formula:

$Volume=(Length\times Width^{2})/2$.

At the endpoint of the experiment, major organs including the heart, liver, spleen, lungs, and kidneys were collected for histological examination via hematoxylin and eosin (H&E) staining. Blood samples were also collected for liver function tests (ALT/AST) and complete blood panel analysis.


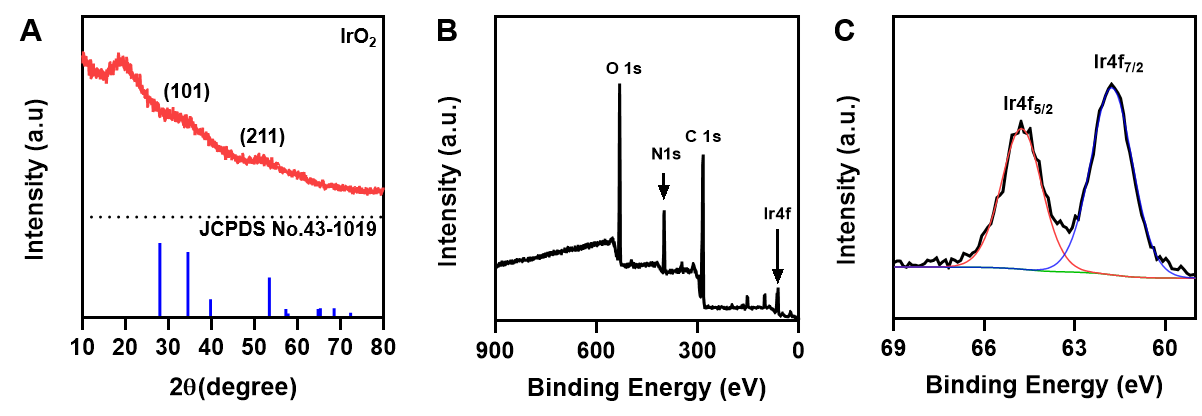


**Figure S****1.** Phase identification of the as-synthesized IrO₂ nanocore. **(A)** X-ray diffraction (XRD) pattern of IrO₂ exhibiting reflections at 2θ ≈ 34° and 53°, indexed to the (101) and (211) planes of rutile-phase IrO₂ (JCPDS No. 43-1019), respectively. **(B)** XPS survey spectrum of pristine IrO₂, resolving characteristic Ir 4f, O 1s, N 1s and C 1s photoelectron signals. **(C)** High-resolution Ir 4f spectrum of pristine IrO₂ showing the well-resolved doublet peaks at 61.7 eV (Ir 4f₇/₂) and 64.7 eV (Ir 4f₅/₂), characteristic of the Ir⁴⁺ oxidation state and fully consistent with rutile-phase IrO₂.


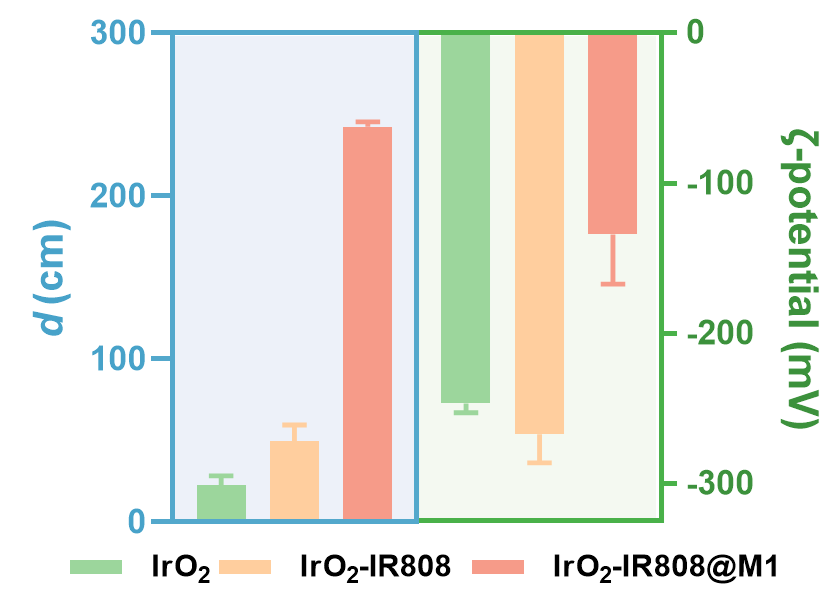


**Figure S2.** Hydrodynamic-size and ζ-potential of across the three fabrication stages of the nanoplatform as measured by dynamic light scattering. Data are mean ± SD of three independent assays.


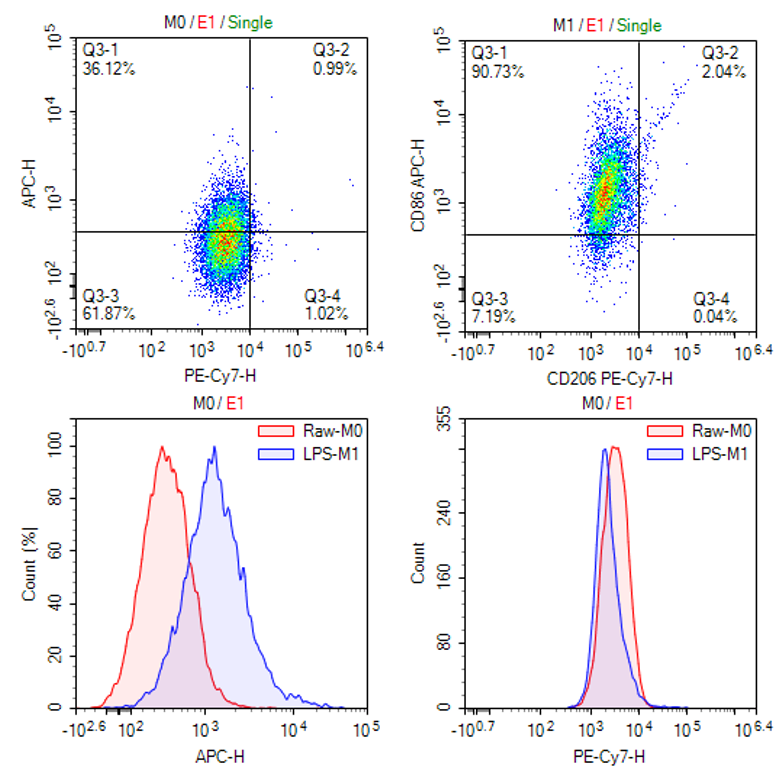


**Figure S3.** Verification of M1 polarisation of RAW264.7 macrophages by paired flow-cytometric profiling of the canonical M1 (CD86) and M2 (CD206) surface markers. Representative CD86 fluorescence histograms of unstimulated (grey) and LPS (100 ng mL⁻¹) + IFN-γ (20 ng mL⁻¹)-co-stimulated (red) RAW264.7 cells (24 h stimulation); the CD86⁺ population increased from 36.12 % to 90.73 % upon stimulation. Representative CD206 fluorescence histograms of the same paired groups show concomitant downregulation of CD206. Cells were stained with PE-anti-CD86 and APC-anti-CD206 antibodies and acquired on a BD FACS Calibur cytometer; ≥ 1 × 10⁴ events were collected per sample. Data are representative of three independent biological replicates.


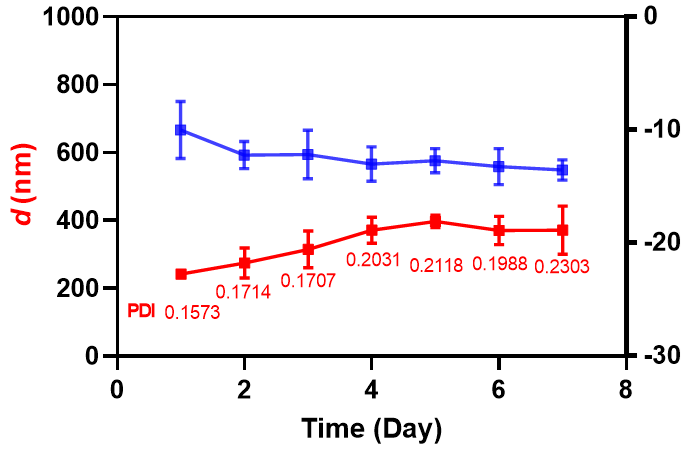


**Figure S4.** Colloidal stability of IrO₂-IR808@M1. Data are mean ± SD; *n* = 3 independent measurements per time point.


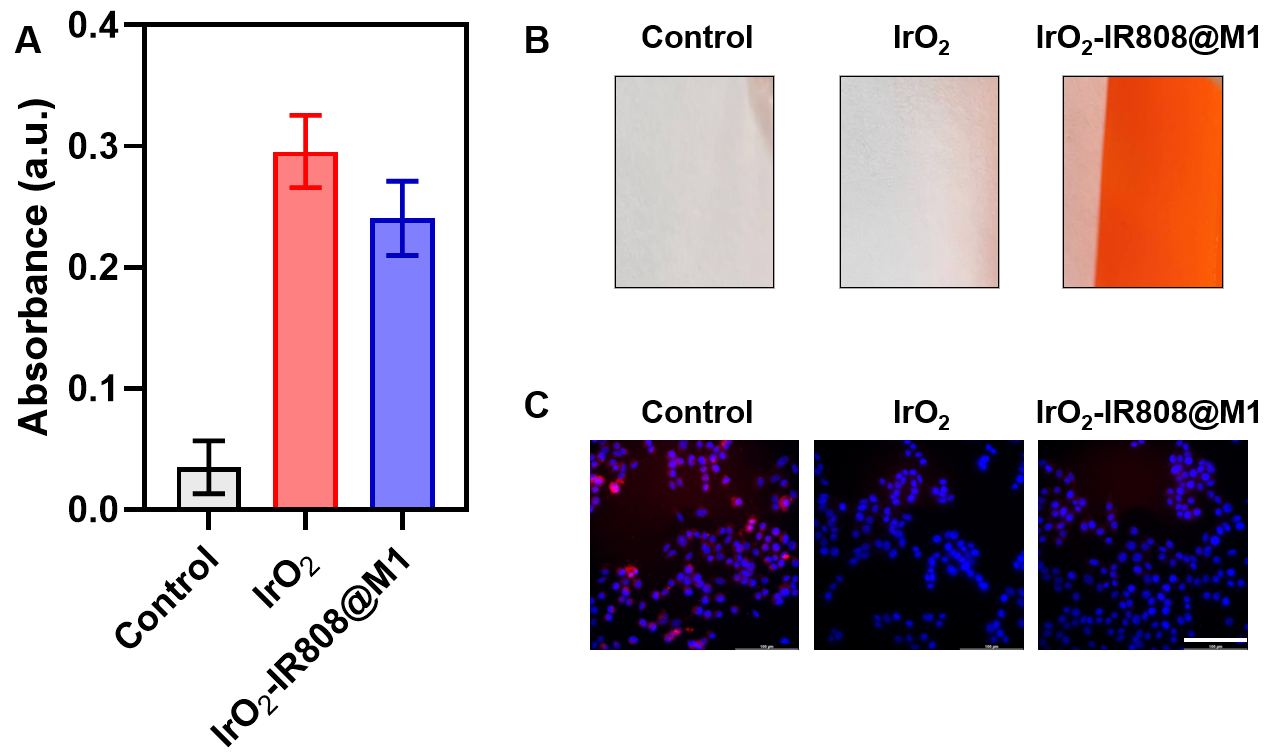


**Figure S5.** Three orthogonal assays validating the catalase-mimicking, oxygen-self-supplying activity of the nanoplatform. **(A)** 3,3′,5,5′-Tetramethylbenzidine (TMB) chromogenic assay: incubation of IrO₂ or IrO₂-IR808@M1 with H₂O₂ catalysed the oxidation of TMB to the blue-coloured oxidised product (oxTMB), yielding characteristic absorbance at 652 nm of 0.29 ± 0.03 (IrO₂) and 0.24 ± 0.03 (IrO₂-IR808@M1) *versus* 0.04 ± 0.02 for the control (mean ± SD, *n* = 3). **(B)** Photographs of macroscopic gas-bubble evolution within seconds of H₂O₂ addition to the IrO₂ and IrO₂-IR808@M1 vials, in contrast to the bubble-free control. **(C)** Fluorescence images of the oxygen-sensitive phosphorescent probe [Ru(dpp)₃]Cl₂ (1 µM), whose red signal is dynamically quenched by molecular O₂; markedly attenuated fluorescence was observed in the IrO₂ and IrO₂-IR808@M1 groups relative to the control, providing direct in situ evidence of O₂ release. Scale bar: 100 µm.

**Figure S6.** Power-density-resolved photothermal benchmarking of IrO₂ alone. Temperature elevation profiles of an aqueous IrO₂ suspension (same concentration and irradiation geometry as used for IrO₂-IR808@M1 in Figure 2B) under 808 nm continuous-wave laser irradiation for 15 min at power densities of 1.0, 1.5 and 2.0 W cm⁻². Temperature was recorded every 30 s using an FLIR thermal camera. Side-by-side comparison with the IrO₂-IR808@M1 curves of Figure 2B demonstrates that IrO₂-IR808@M1 outperforms IrO₂ across the full power-density range, supporting the synergistic photothermal contribution of IR808. Data are mean ± SD; *n* = 3 independent measurements per power density.

**Figure S7.** Quantitative cellular-uptake kinetics of rhodamine-B-labelled IrO₂-IR808@M1 in MCF-7 cells. Rhodamine-B channel was quantified from confocal images at 30 min, 1 h, 2 h, 4 h, 8 h and 24 h post-incubation. At each time point, ≥ 5 randomly selected fields and ≥ 50 cells per field were analyzed. Data are presented as mean ± SD.

**Figure S8.** Hematological profiling of 4T1-tumor-bearing BALB/c mice following IrO₂-IR808@M1 treatment. Whole-blood parameters including white blood cell count (WBC), red blood cell count (RBC), haemoglobin (HGB), haematocrit (HCT), mean corpuscular volume (MCV), mean corpuscular haemoglobin (MCH), mean corpuscular haemoglobin concentration (MCH) and platelet count (PLT) were measured 15 days after the start of the treatment course (PBS, IrO₂ + NIR, and IrO₂-IR808@M1 + NIR groups). All parameters remained within the clinically normal reference ranges across the three groups, indicating absence of hematological toxicity. Data are mean ± SD; *n* = 3 mice per group
